# New Diagnostic and Therapeutic Developments in Eye and Systemic Diseases

**DOI:** 10.3390/life16071196

**Published:** 2026-07-20

**Authors:** Zhihong Jewel Hu

**Affiliations:** Doheny Image Analysis Laboratory, Doheny Eye Institute, 150 North Orange Grove Blvd, Pasadena, CA 91103, USA; jhu@doheny.org

The rapid evolution of diagnostic and therapeutic technologies continues to reshape ophthalmology and its interface with systemic diseases. This Special Issue, “New Diagnostic and Therapeutic Developments in Eye and Systemic Diseases,” highlights clinically grounded advances in ocular structure and function and the link with systemic health and disease management. The nine contributions in this first edition provide concrete examples of how emerging technologies and therapies are being translated into clinical and research settings.

In the field of retinal disease, such as age-related macular degeneration (AMD), multiple studies address both diagnosis and management. An artificial intelligence (AI) deep learning framework (Sliding Window Attention U-Net: SWAU-Net) introduces a hybrid Transformer-CNN (convolutional neural network) model capable of predicting longitudinal geographic atrophy progression, offering potential for personalized monitoring and clinical trial optimization [1]. Complementing this, a practical image-processing approach using grayscale inversion significantly enhances visualization of macular neovascularization and polypoidal lesions on optical coherence tomography angiography (OCTA), improving clinical interpretability without requiring new hardware [2].

Therapeutic advancements of retinal disease are highlighted by two real-world studies evaluating high-dose 8 mg aflibercept in treatment-refractory neovascular AMD [3,4]. Across cohorts with extensive prior anti-VEGF (Vascular Endothelial Growth Factor) exposure, switching to 8 mg aflibercept was primarily driven by persistent fluid or the need for extended treatment intervals. The studies demonstrate improved treatment durability, interval extension, and anatomical benefits such as reduced pigment epithelial detachment, while maintaining stable visual acuity and a favorable short-term safety profile.

Beyond retinal disease, this Special Issue also addresses surgical and refractive outcomes in patients with ocular and systemic comorbidities. A study on cataract surgery demonstrates that a standardized regimen with the combination of intracameral mydriatics and anesthetic (SCIMA) maintains stable mydriasis in over 90% of patients, including those with diabetes and pseudoexfoliation syndrome, supporting its safety in complex clinical settings [5]. Visual rehabilitation is further explored through bilateral implantation of an enhanced monofocal intraocular lens using a mini-monovision approach, which resulted in excellent distance and intermediate visual acuity and high patient satisfaction [6]. Similarly, single-step transepithelial photorefractive keratectomy (TPRK) achieved highly predictable refractive outcomes, with 96% of eyes reaching 20/20 vision or better and no significant safety concerns [7].

At the level of systemic and metabolic influences, a review on taurine and histidine highlights their potential neuroprotective and metabolic roles in retinal diseases such as diabetic retinopathy, glaucoma, and AMD, while emphasizing the current lack of robust clinical validation [8]. Another systematic review underscores the impact of long-term glaucoma therapy on the ocular surface, demonstrating that preservative-free formulations significantly improve tolerability and may reduce the burden of ocular surface disease in chronic treatment [9].

Across these studies, several common themes emerge. First, advances in imaging and AI are enabling more precise, quantitative, and predictive assessment of disease. Second, real-world evidence is refining therapeutic strategies, particularly in chronic retinal diseases requiring long-term management. Third, patient-centered outcomes, such as treatment burden, surgical safety, and quality of life, are increasingly prioritized. However, challenges remain, including limited standardization, variability in measurement techniques, and the need for larger, longitudinal studies to validate emerging biomarkers and therapies.

In summary, this Special Issue moves beyond general concepts by providing specific methodological and clinical insights from AI-driven disease prediction and simple image enhancement techniques to optimized pharmacologic and surgical interventions. Together, these contributions advance a more integrated and practical approach to diagnosing and managing eye diseases within the broader context of systemic health, and they lay a foundation for future work in precision and translational ophthalmology.
